# Dropout in Neural Networks Simulates the Paradoxical Effects of Deep Brain Stimulation on Memory

**DOI:** 10.3389/fnagi.2020.00273

**Published:** 2020-09-14

**Authors:** Shawn Zheng Kai Tan, Richard Du, Jose Angelo Udal Perucho, Shauhrat S. Chopra, Varut Vardhanabhuti, Lee Wei Lim

**Affiliations:** ^1^Neuromodulation Laboratory, School of Biomedical Sciences, Li Ka Shing Faculty of Medicine, The University of Hong Kong, Hong Kong, Hong Kong; ^2^Department of Diagnostic Radiology, Li Ka Shing Faculty of Medicine, The University of Hong Kong, Hong Kong, Hong Kong; ^3^School of Energy and Environment, City University of Hong Kong, Hong Kong, Hong Kong

**Keywords:** neuromodulation, deep brain stimulation, memory, neural network, dropout

## Abstract

Neuromodulation techniques such as deep brain stimulation (DBS) are a promising treatment for memory-related disorders including anxiety, addiction, and dementia. However, the outcomes of such treatments appear to be somewhat paradoxical, in that these techniques can both disrupt and enhance memory even when applied to the same brain target. In this article, we hypothesize that disruption and enhancement of memory through neuromodulation can be explained by the dropout of engram nodes. We used a convolutional neural network (CNN) to classify handwritten digits and letters and applied dropout at different stages to simulate DBS effects on engrams. We showed that dropout applied during training improved the accuracy of prediction, whereas dropout applied during testing dramatically decreased the accuracy of prediction, which mimics enhancement and disruption of memory, respectively. We further showed that transfer learning of neural networks with dropout had increased the accuracy and rate of learning. Dropout during training provided a more robust “skeleton” network and, together with transfer learning, mimicked the effects of chronic DBS on memory. Overall, we showed that the dropout of engram nodes is a possible mechanism by which neuromodulation techniques such as DBS can both disrupt and enhance memory, providing a unique perspective on this paradox.

## Introduction

Memory systems are crucial for survival and, to a large extent, define who we are. However, memory systems can fall into disease when expressed pervasively (e.g., anxiety or addiction) or degenerate (e.g., dementia)—both of which are major health challenges worldwide (World Health Organization, 2012, 2017). Neuromodulation techniques such as deep brain stimulation (DBS) have shown promising results as treatments for memory-related disorders (Tan et al., 2019b, 2020b), yet the mechanisms behind these effects are still largely unknown. Furthermore, the effects of treatments such as DBS appear to be paradoxical, in that they can both disrupt (Hamani et al., 2010; Tan et al., 2019a) and enhance memories (Hamani et al., 2011; Tan et al., 2020c) even when applied to the same brain target (detailed review in Tan et al., 2020b). We have previously suggested that DBS is able to disrupt memory by “removing” nodes in an engram (Tan et al., 2019b). Engrams are a theoretical means by which memory is physically stored in the brain and can be thought of as a subset of neurons in a given memory brain region (e.g., hippocampus) that are recruited in the initial memory encoding phase (Ramirez et al., 2013). In this manuscript, we take the view that engrams are plastic synapses (associative/connectionist model), and hence, engram nodes are synapses between engram neurons (Langille and Gallistel, 2020). Despite increased knowledge of engrams and new techniques to study them, the theory that DBS disrupts memory by “removing” engram nodes (synapses associated with the engram) remains untested partly due to the lack of technology to monitor large engram networks in real time. Besides, this theory does not explain (at least directly) how memory enhancement is achieved.

The development of machine learning techniques offers a unique computational approach to simulate hypothetical models of learning and memory, and the effects of manipulation on memory, which we have previously used to highlight potential mechanisms of memory disruption by DBS (Tan et al., 2019b). To model the learning process, we trained a convolutional neural network (CNN) to classify handwritten digits and letters.

CNNs are a type of artificial neural network that is commonly applied to image recognition tasks. The concept of CNNs was developed from early observations of the visual cortex by Hubel and Wiesel (1959, 1962), in which groups of neurons fired distinctively in response to different light patterns (e.g., straight lines, circles). In a typical CNN, features are extracted by the network using a convolutional layer followed by classification. For image tasks, this convolutional layer is comprised of a series of convolution filters that are associated with particular patterns of pixels, which mimic receptive fields in the retina. These trainable filters are also referred to as weights of the network, similar to synapse/synaptic strength in biological systems. In image classification, the CNN decomposes the input image into patterns of pixels known as features. First, an input image is partitioned into non-overlapping regions with each region mapped to a specific neuron. Second, the neurons are convolved by multiple filters to generate a feature map in the convolution layer. The resultant feature maps can be further decomposed by inputting these maps into successive convolution layers. After a specified number of decompositions, the resultant features are used to classify the input image using the fully connected layer.

In this article, we hypothesize that the paradoxical ability of DBS to both disrupt and enhance memory can be explained through dropout (a process of randomly shutting down or dropping neurons) in engram nodes by using CNN to simulate learning and memory.

Due to limited systematic studies looking at DBS and memory, we based our modeling on the results from our previous studies that showed high-frequency stimulation of the ventromedial prefrontal cortex (vmPFC) could both enhance (Tan et al., 2020c) and disrupt (Tan et al., 2019a) memory. Based on these findings, we focused on the hippocampal engram and implicit associative memories in the proposed simulation.

## Materials and Methods

### Dataset

To model the learning process, we trained a CNN to classify handwritten digits and letters from the EMNIST dataset. The EMNIST dataset is a public database of over 800,000 handwritten digits and letters across 62 different classes (Cohen et al., 2017). In our study, we used the EMNIST balanced dataset, which is derived by merging similar classes of letters. This dataset contains 131,600-28 × 28 pixel images of 47 balanced classes (10 digits and 37 uppercase and lowercase letters).

### Network Architecture

The CNN consists of an input layer of size 28 × 28 × 1 followed by two convolution layers of 32 and 64 filters, leading to a feature map of size 5 × 5 × 64. No padding was used for the convolutions, and a filter of size 3 × 3 was used in both layers. At the end of each convolution layer, we applied max pooling with a 2 × 2 window. Max pooling is a standard process for reducing the dimensions of feature maps by sampling the maximum value for a given window size, which forces the network to enhance and focus on important features (Yamaguchi et al., 1990). Global max pooling was applied to the feature map to extract 64 × 1 latent features. For feature classification, the features were passed through to a fully connected layer of 100 neurons. A rectified linear unit (ReLU) was applied as an activation function in all the layers, which is a ramp function where all negative value neurons are zeroed to ensure unidirectionality. The parameters in all the networks were optimized using binary cross-entropy or loss function. All networks were trained for 500 epochs in batches of 10 images. An epoch is defined as one complete run-through of all the training data. For the analysis, the average loss of training data and average classification accuracy of the testing data were evaluated at the end of each epoch.

### Experiments

**Figure 1:**
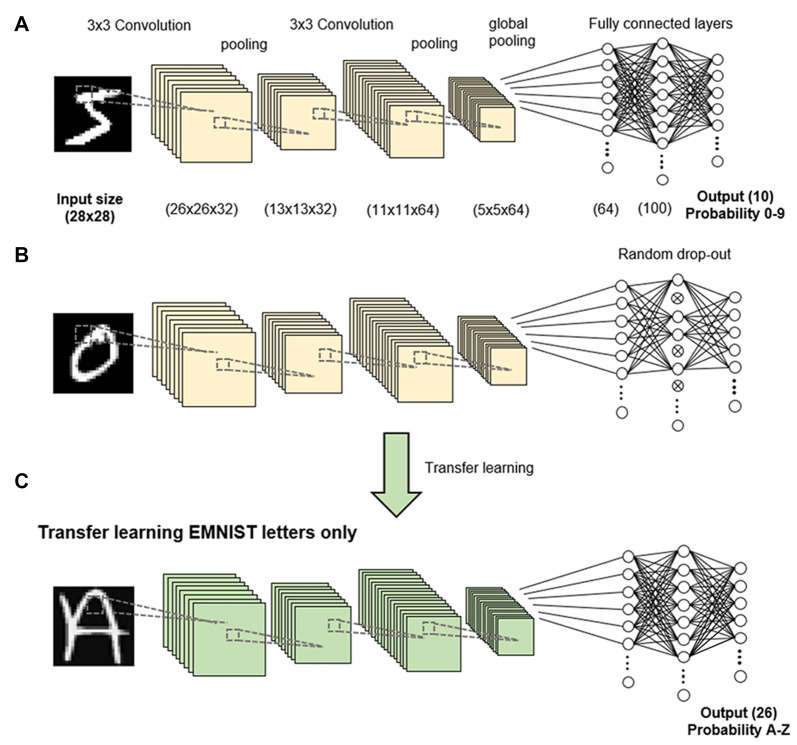
Methodology. A convolutional neural network (CNN) was trained to classify handwritten digits in the EMNIST dataset **(A)**. Dropout was applied to 50% of nodes in the fully connected layer **(B)**. Transfer learning was performed to retrain the network to recognize uppercase letters **(C)**.

We conducted three sets of experiments in this study. In Experiment 1, we trained a network to classify 10 different digits (0–9) from the EMNIST balanced dataset. We applied dropout (a process of randomly shutting down or dropping neurons) either during the training stages or just prior to testing at each epoch. We used a dropout rate of 50%, such that half the neurons were dropped at each step (Figures 1A,B). Dropout was only done on fully connected latter layers of the network. As the aim of the study was not to train a network to classify the digits accurately but to analyze the learning process, the network was trained on 1,000 randomly sampled digits as the training dataset, and the network was evaluated on another 1,000 randomly sampled digits as the testing dataset. All networks were trained with the same training dataset and tested with the same testing dataset.

In Experiment 2, we similarly trained a network identical to that of Experiment 1 but applied variable dropout rates (20%, 40%, 60%, and 80%) during the training stages.

In Experiment 3, we transferred the network to learn 26 uppercase letters (A to Z) in a process known as transfer learning, in which the networks and weights of the control (non-dropout) group and dropout group were applied to the new task (Figure 1C). We retrained the network to recognize uppercase letters by stripping the last output layer and replacing it with the 26 classes corresponding to each letter class. We evaluated the performance of the transfer learning using the trained network with and without dropout compared to the performance of a network directly trained on letters without transfer learning (Figure 1C). Due to the increased complexity of more letter classes, we used 5,000 randomly sampled letters as the training dataset and 1,000 randomly sampled letters as the testing dataset.

## Results

### Experiment 1

**Figure 2:**
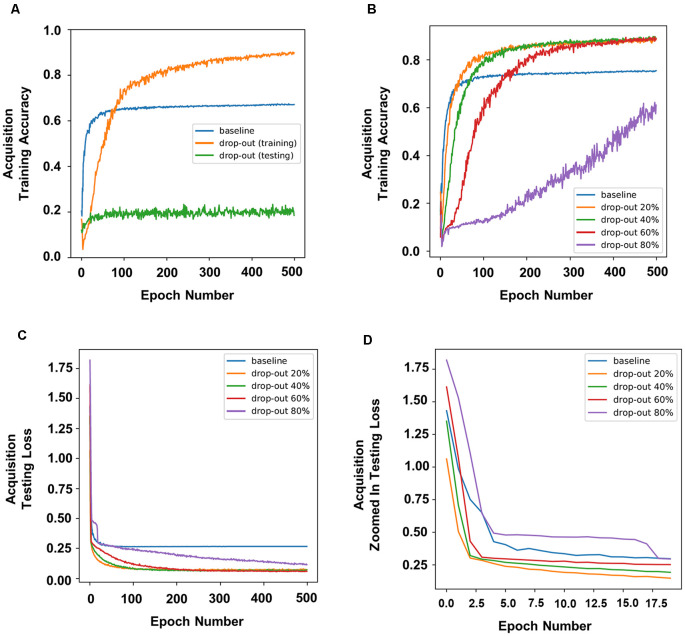
Dropout in neural nodes disrupted or enhanced learning in the neural networks depending on when it was applied. Dropout applied during training improved prediction accuracy, whereas dropout applied during testing dramatically decreased prediction accuracy **(A)**. **(B)** Prediction accuracy of different rates of dropout during training. **(C)** The training loss and **(D)** a zoomed in version of the first 20 epochs in panel **(C)**.

To simulate the effects of DBS on memory, we applied dropout during the training or testing stages. The accuracy of prediction was used as an indication of how “well” the neural network had learned the task, which serves as a proxy for memory. Therefore, a higher accuracy of prediction should indicate higher memory function. In our experiment, dropout applied during training improved the accuracy of prediction, whereas dropout applied during testing dramatically decreased the accuracy of prediction (Figure 2A).

### Experiment 2

To simulate the different parameters of DBS and to further understand how different rates of dropout would affect DBS during the training stages, we applied different percentages of dropout. In our experiments, a dropout of 20%, 40%, or 60% resulted in a higher training accuracy, whereas a dropout of 80% resulted in a lower training accuracy (Figure 2B). Interestingly, 20% or 40% dropout resulted in lower training loss (defined as the average errors made across the testing) than the baseline initially, and 60% dropout resulted in higher training loss than the baseline by the second epoch, whereas 80% dropout showed the highest training loss throughout (Figures 2C,D).

### Experiment 3

**Figure 3:**
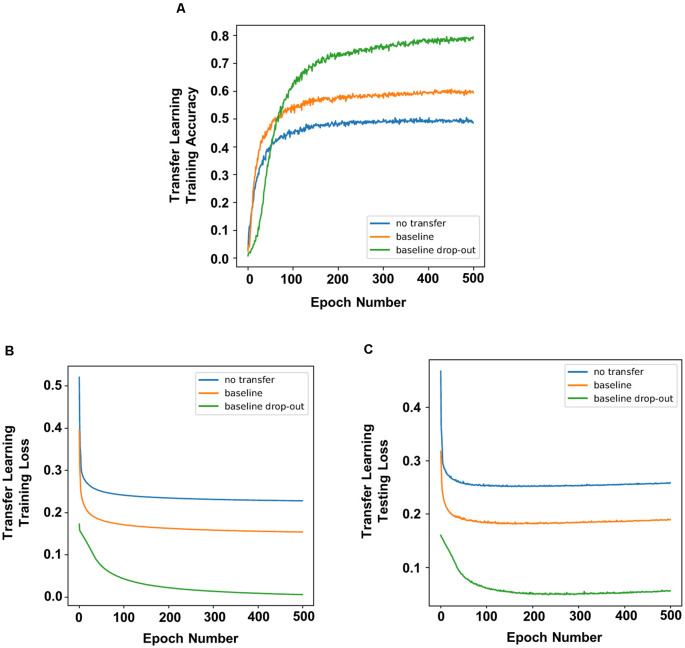
Transfer learning of networks that underwent dropout showed higher prediction accuracy and faster learning. Compared to non-dropout networks, transfer learning of neural networks with dropout had increased accuracy **(A)** with lower training loss **(B)** and testing loss **(C)**.

To simulate a novel memory task post chronic DBS, we applied the process of transfer learning on the trained network, in which the networks and weights of the control (non-dropout) group and dropout groups were applied to a new task. Overall, transfer learning of a network showed increased accuracy compared to a network without transfer learning (Figure 3A), indicating higher memory function. In addition, there were decreased testing and training losses (Figures 3B,C; defined as the average errors made across the testing or training, respectively). Decreased loss indicates better performance or better fitting of the training/testing data, which serves as a proxy for increased rate of learning. Transfer learning in neural networks with dropout resulted in better accuracy (Figure 1) and lower training and testing loss (Figures 3B,C) compared to transfer learning in networks with no dropout.

## Discussion

We have previously argued that timing plays an important role in the outcome of DBS on memory (Tan et al., 2020b). DBS applied post or during behavior testing tended to disrupt memory, whereas DBS applied prior to behavior testing tended to enhance memory. However, the mechanisms behind these outcomes are still relatively unknown. Indeed, we found that dropout applied during training improved the accuracy of prediction, which was similar to the enhancement of memory seen when DBS was applied prior to behavior testing in our previous animal studies (Liu et al., 2015). On the contrary, we found that dropout applied post training dramatically decreased the accuracy of prediction, which was similar to the disruption of memory seen when DBS was applied during consolidation of memory in our previous animal studies (Tan et al., 2019a; Figure 2A).

We used CNNs (as opposed to standard fully connected networks) in this study as they are commonly applied in image recognition tasks. Although CNNs are modeled on the visual cortex, dropout was only applied on fully connected latter layers in the network to represent dropouts applied in the hippocampus rather than in the visual cortex. This mimicked our previous experiments on the effects of prefrontal cortex stimulation on the hippocampus (Tan et al., 2019a, 2020c).

In order to simulate different DBS parameters in a generalized manner, we applied different percentage dropouts during the training stage. We showed that lower dropout rates (20%) effectively increased both accuracy and training loss, whereas a higher dropout rate (80%) drastically decreased the ability of the neural network to learn the task effectively. First, this indicates that DBS is more likely to result in lower dropout rates. Second, it simulates variations in DBS parameters in a generalized way, showing that even at low dropout rates (20%), the effects are still robust, and is only ineffective at high dropout rates.

One difference between Experiment 1 and our previous animal experiments was that chronic DBS was not applied during the training stage, but rather in the home-cage prior to behavior experiments. To more accurately represent the enhancement of memory through chronic DBS prior to a memory task (Liu et al., 2015), we applied transfer learning in previously trained networks with or without dropout. We showed that transfer learning did indeed increase the accuracy of prediction and decrease training and testing loss (representing an increased learning rate), indicating that transfer learning was successful in this model. More importantly, we showed that transfer learning applied to neural networks with dropout increased the accuracy of prediction, indicating higher memory function compared to neural networks without dropout. We further showed that transfer learning applied to neural networks with dropout had lower training and testing loss, indicating not only improved memory function but also increased rate of learning. Overall, we showed that applying dropout during training provides a more robust “skeleton” network, and applying transfer learning in this network increases accuracy and decreases training and testing loss. This model showed similar memory enhancement results to that in our previous study on chronic mPFC DBS (Liu et al., 2015), which suggests a potential mechanism for this process.

An early hypothesis of the mechanism of DBS was that it creates a temporary neural activity lesion (McIntyre et al., 2004). We further showed that prelimbic cortex DBS was associated with the disruption of memory and a drop in the neural activity marker c-fos in the ventral hippocampus (Tan et al., 2019a). Dropout of neural nodes, while congruent with the disruption of memory, has not played a major role in the mechanistic understanding of memory enhancement seen in DBS, although it should be noted that other mechanisms such as neurogenesis and wave syncing have been suggested (Tan et al., 2020b). In this article, we propose that DBS causes dropout in neural nodes that “forces” the activation of new pathways and creates more robust networks, similar to how dropout enhanced the neural networks. Mechanistically in the brain, this could be hypothesized to relate to increased synaptic plasticity or synaptogenesis—activating new pathways could be seen as the formation of new synapses, and the change in weights by the “backward steps” of dropout could be related to synaptic plasticity. Indeed, DBS has been shown to be related to both plasticity and synaptogenesis (Visanji et al., 2015; Pohodich et al., 2018). It is, however, difficult to properly hypothesize how DBS achieves this mechanistically due to the complexities of the changes induced by DBS and the fact we still do not know how it works. To the best of our knowledge, this article is the first to suggest a cohesive mechanism in which disruption of neural activity through DBS can lead to both disruption and enhancement of memory. Although no behavioral experiments were performed in conjunction with our modeling, we based the models on our previous animal behavioral experiments that showed that DBS prior to the behavioral task resulted in memory enhancement (Tan et al., 2020c) and DBS during consolidation of memory resulted in memory disruption (Tan et al., 2019a), which were similarly demonstrated in this present article. However, we acknowledge the simplistic and preliminary nature of our methodology and results. Although more complex models of associative memory and learning have been previously presented (Yusoff and Grüning, 2012; Osawa and Kohno, 2015), the lack of understanding on how neuromodulation actually affects neuronal firing in memory, especially in a remote downstream target like the hippocampus (in the case of vmPFC neuromodulation), makes it difficult to use these models. A simpler model, like that one presented here, is therefore more generalizable and better serves as a hypothetical and conceptual tool for more sophisticated research.

Although DBS has been suggested as a treatment for memory-related disorders including Alzheimer’s disease (AD) and anxiety disorders, its mechanism is still largely unknown. In this article, we suggest hypothetical mechanisms for the effects of DBS on memory. We showed that dropout of engram nodes could disrupt memory processes, which might be useful in translating DBS as a treatment for anxiety disorders like PTSD (Gouveia et al., 2020). We also showed that dropout of engram nodes could improve memory function and suggested that this happens through increased synaptic function. Given that impaired synaptic plasticity and synaptogenesis have been associated with AD (Levi et al., 2003; Shankar et al., 2008) and DBS has shown promise as a treatment for AD (Liu et al., 2015), dropout of engram nodes could be a potential mechanism of DBS neuromodulation.

In conclusion, using a machine learning model based on previous animal experiments, we showed that dropout of nodes could be a potential mechanism in which neuromodulation techniques like DBS can both disrupt and enhance memory. While preliminary in nature, this article serves as a basis for further experimentation on engrams to understand the effects of neuromodulation on memory.

## Data Availability Statement

Publicly available datasets were analyzed in this study. This data can be found here: https://www.nist.gov/itl/products-and-services/emnist-dataset.

## Author Contributions

ST, RD, and SC conceived and designed the experiments. JP, LWL, and VV contributed significantly to the experimental design. RD conducted the experiments. ST, RD, and LWL contextualized the data. ST drafted the manuscript. All authors contributed to the article and approved the submitted version.

## Disclaimer

Frontiers Media SA and the authors remain neutral with regard to jurisdictional claims in published maps and institutional affiliations.

## Conflict of Interest

The authors declare that the research was conducted in the absence of any commercial or financial relationships that could be construed as a potential conflict of interest.

## References

[B1] Cohen G. Afshar S. Tapson J. van Schaik A. (2017). “EMNIST: an extension of MNIST to handwritten letters,” in 2017 International Joint Conference on Neural Networks (IJCNN), Anchorage, AK, pp. 2921–2926.

[B2] Gouveia F. V. Davidson B. Meng Y. Gidyk D. C. Rabin J. S. (2020). Treating post-traumatic stress disorder with neuromodulation therapies: transcranial magnetic stimulation, transcranial direct current stimulation and deep brain stimulation. Neurotherapeutics [Epub ahead of print]. 10.1007/s13311-020-00871-0 32468235 PMC7851279

[B3] Hamani C. Dubiela F. P. Soares J. C. K. Shin D. Bittencourt S. Covolan L.. (2010). Anterior thalamus deep brain stimulation at high current impairs memory in rats. Exp. Neurol. 225, 154–162. 10.1016/j.expneurol.2010.06.007 20558163

[B4] Hamani C. Stone S. S. Garten A. Lozano A. M. Winocur G. (2011). Memory rescue and enhanced neurogenesis following electrical stimulation of the anterior thalamus in rats treated with corticosterone. Exp. Neurol. 232, 100–104. 10.1016/j.expneurol.2011.08.023 21906593

[B5] Hubel D. H. Wiesel T. N. (1959). Receptive fields of single neurones in the cat’s striate cortex. J. Physiol. 148, 574–591. 10.1113/jphysiol.1959.sp006308 14403679 PMC1363130

[B6] Hubel D. H. Wiesel T. N. (1962). Receptive fields, binocular interaction and function architecture in the cat’s visual cortex. J. Physiol. 160, 106–154. 10.1113/jphysiol.1962.sp006837 14449617 PMC1359523

[B7] Langille J. J. Gallistel C. R. (2020). Locating the engram: should we look for plastic synapses or information-storing molecules? Neurobiol. Learn. Mem. 169:107164. 10.1016/j.nlm.2020.107164 31945459

[B8] Levi O. Jongen-Relo A. L. Feldon J. Roses A. D. Michaelson D. M. (2003). ApoE4 impairs hippocampal plasticity isoform-specifically and blocks the environmental stimulation of synaptogenesis and memory. Neurobiol. Dis. 13, 273–282. 10.1016/s0969-9961(03)00045-7 12901842

[B9] Liu A. Jain N. Vyas A. Lim L. W. (2015). Ventromedial prefrontal cortex stimulation enhances memory and hippocampal neurogenesis in the middle-aged rats. elife 4:e04803. 10.7554/elife.04803 25768425 PMC4381300

[B10] McIntyre C. C. Savasta M. Kerkerian-Le Goff L. Vitek J. L. (2004). Uncovering the mechanism(s) of action of deep brain stimulation: activation, inhibition, or both. Clin. Neurophysiol. 115, 1239–1248. 10.1016/j.clinph.2003.12.024 15134690

[B11] Osawa Y. Kohno T. (2015). Associative memory with class I and II Izhikevich Model. J. Robot. Netw. Artif. Life 1:312 10.2991/jrnal.2015.1.4.12

[B12] Pohodich A. E. Yalamanchili H. Raman A. T. Wan Y.-W. Gundry M. Hao S.. (2018). Forniceal deep brain stimulation induces gene expression and splicing changes that promote neurogenesis and plasticity. elife 7:e34031. 10.7554/elife.34031 29570050 PMC5906096

[B13] Ramirez S. Liu X. Lin P.-A. Suh J. Pignatelli M. Redondo R. L.. (2013). Creating a false memory in the hippocampus. Science 341, 387–391. 10.1126/science.1239073 23888038

[B14] Shankar G. M. Li S. Mehta T. H. Garcia-Munoz A. Shepardson N. E. Smith I.. (2008). Amyloid-β protein dimers isolated directly from Alzheimer’s brains impair synaptic plasticity and memory. Nat. Med. 14, 837–842. 10.1038/nm1782 18568035 PMC2772133

[B15] Tan S. Z. K. Du R. Perucho J. A. U. Chopra S. S. Vardhanabhuti V. Lim L. W. (2020a). Dropout in neural networks simulates the paradoxical effects of deep brain stimulation on memory. bioRxiv [Preprint]. 10.1101/2020.05.01.073486 PMC7521073 33093830

[B16] Tan S. Z. K. Fung M. Koh J. Chan Y. Lim L. W. (2020b). The paradoxical effect of deep brain stimulation on memory. Aging Dis. 11, 179–190. 10.14336/ad.2019.0511 32010491 PMC6961776

[B1600] Tan S. Z. K. Neoh J. Lawrence A. J. Wu E. X. Lim L. W. (2020c). Prelimbic cortical stimulation improves spatial memory through distinct patterns of hippocampal gene expression in aged rats. Neurotherapeutics 10.1007/s13311-020-00913-7 32816221 PMC7851284

[B17] Tan S. Z. K. Poon C. H. Chan Y.-S. Lim L. W. (2019a). Deep brain stimulation of the ventromedial prefrontal cortex disrupts consolidation of fear memories. bioRxiv [Preprint]. 10.1101/537514

[B18] Tan S. Z. K. Sheng V. Chan Y.-S. Lim L. W. (2019b). Eternal sunshine of the neuromodulated mind: altering fear memories through neuromodulation. Exp. Neurol. 314, 9–19. 10.1016/j.expneurol.2019.01.004 30639183

[B19] Visanji N. P. Sarvestani I. K. Creed M. C. Shoaei Z. S. Nobrega J. N. Hamani C. (2015). Deep brain stimulation of the subthalamic nucleus preferentially alters the translational profile of striatopallidal neurons in an animal model of parkinson’s disease. Front. Cell. Neurosci. 9:221 10.3389/fncel.2015.00221 26106299 PMC4460554

[B20] World Health Organization (2012). Dementia cases set to triple by 2050 but still largely ignored [WWW Document]. Available online at: http://www.who.int/mediacentre/news/releases/2012/dementia_20120411/en/. Accessed June 11, 2020

[B21] World Health Organization (2017). Depression and other common mental disorders: global health estimates. Available online at: https://www.who.int/mental_health/management/depression/prevalence_global_health_estimates/en/. Accessed August 31, 2020.

[B22] Yamaguchi K. Sakamoto K. Akabane T. Fjuimoto Y. (1990). “A neural network for speaker-independent isolated word recognition,” in ISCA Arch (Kobe, Japan), 1077–1080.

[B23] Yusoff N. Grüning A. (2012). Biologically inspired temporal sequence learning. Procedia Eng. 41, 319–325. 10.1016/j.proeng.2012.07.179

